# Ablation of *Mrds1/Ofcc1* Induces Hyper-γ-Glutamyl Transpeptidasemia without Abnormal Head Development and Schizophrenia-Relevant Behaviors in Mice

**DOI:** 10.1371/journal.pone.0029499

**Published:** 2011-12-29

**Authors:** Tetsuo Ohnishi, Kazuo Yamada, Akiko Watanabe, Hisako Ohba, Toru Sakaguchi, Yota Honma, Yoshimi Iwayama, Tomoko Toyota, Motoko Maekawa, Kazutada Watanabe, Sevilla D. Detera-Wadleigh, Shigeharu Wakana, Takeo Yoshikawa

**Affiliations:** 1 Laboratory for Molecular Psychiatry, RIKEN Brain Science Institute, Wako, Japan; 2 Department of Bioenginering, Nagaoka University of Technology, Nagaoka, Japan; 3 Mood and Anxiety Disorders Program, National Institute of Mental Health, Bethesda, Maryland, United States of America; 4 Technology and Development Team for Mouse Phenotype Analysis, RIKEN BioResource Center, Tsukuba, Japan; University of Queensland, Australia

## Abstract

Mutations in the *Opo* gene result in eye malformation in medaka fish. The human ortholog of this gene, *MRDS1/OFCC1*, is a potentially causal gene for orofacial cleft, as well as a susceptibility gene for schizophrenia, a devastating mental illness. Based on this evidence, we hypothesized that this gene could perform crucial functions in the development of head and brain structures in vertebrates. To test this hypothesis, we created *Mrds1/Ofcc1*-null mice. Mice were examined thoroughly using an abnormality screening system referred to as “the Japan Mouse Clinic”. No malformations of the head structure, eye or other parts of the body were apparent in these knockout mice. However, the mutant mice showed a marked increase in serum γ-glutamyl transpeptidase (GGT), a marker for liver damage, but no abnormalities in other liver-related measurements. We also performed a family-based association study on the gene in schizophrenia samples of Japanese origin. We found five single nucleotide polymorphisms (SNPs) located across the gene that showed significant transmission distortion, supporting a prior report of association in a Caucasian cohort. However, the knockout mice showed no behavioral phenotypes relevant to schizophrenia. In conclusion, disruption of the *Mrds1/Ofcc1* gene elicits asymptomatic hyper-γ-glutamyl-transpeptidasemia in mice. However, there were no phenotypes to support a role for the gene in the development of eye and craniofacial structures in vertebrates. These results prompt further examination of the gene, including its putative contribution to hyper-γ-glutamyl transpeptidasemia and schizophrenia.

## Introduction

Genetic studies have implicated the *Mrds1/Ofcc1/Opo* gene in multiple developmental processes although its biological function remains largely unknown. Martinez-Morales *et al.* identified a medaka (a small fish similar to zebrafish) mutant, *ojoplano* (*opo*), which is defective in optic cup morphogenesis, and identified the gene responsible for this phenotype. In addition to the defective optic cup, *opo* mutants also show aberrant craniofacial development and deficits in other structures, including the brain. The gene product of medaka *Opo* is strikingly similar to that of the mammalian *MRDS1/OFCC1/Mrds1/Ofcc1* gene [1], suggesting a function in vertebrate eye and craniofacial development.

This gene has been implicated in two human pathological conditions: orofacial cleft and schizophrenia. Davies *et al.* [2] reported *OFCC1* as a potential orofacial cleft susceptibility gene in humans, based on the observation of three unrelated patients with orofacial cleft, all harboring a chromosomal break within or close to the uncharacterized *OFCC1* gene. Schizophrenia is a severe and debilitating mental illness that afflicts approximately 1% of the world population. While the exact genetic mechanisms of disease pathology remain elusive, recent progress in large-scale analyses has isolated a number of potentially predisposing loci or genes. Human chromosome 6p21–24 has repeatedly been identified as a linkage locus for schizophrenia susceptibility [3], [4], [5].

Straub *et al*. analyzed an Irish family cohort, and identified the *DTNBP1* gene [coding for the dystrobrevin binding protein 1 (dysbindin)] from 6p22.3 as a schizophrenia susceptibility gene [6]. Forty nine families in their sample (18% of total) gave a positive linkage signal at 6p24, when the twenty-seven 6p22.3-linked families (10% of total) were omitted from the analysis. This raises the possibility that the locus harbors more than one schizophrenia susceptibility gene [7]. Follow-up analysis by Straub *et al.* on 262 Irish schizophrenic pedigrees identified a second gene, *MRDS1/OFCC1* (encoding orofacial cleft 1 candidate 1) gene, located on 6p24.3, as a potentially novel candidate gene for schizophrenia [8], [9]. The positive association of schizophrenia with *DTNBP1* has been replicated in independent studies [10], [11], [12], [13], [14], [15], [16], [17], [18], [19], [20], but association with the *MRDS1/OFCC1* gene has not been tested in other ethnic populations.

In this study, we first investigated the relationship between *MRDS1/OFCC1* and normal craniofacial and eye development, as well as schizophrenia, by generating and examining mice deficient in the gene corresponding to human *MRDS1/OFCC1* and medaka *opo.* In addition, we tested the hypothesis of genetic association between the *MRDS1* and schizophrenia using family-based association analysis, in a Japanese cohort.

## Methods

### RACE analysis

3′-RACE was performed using Mouse 15-day Embryo Marathon-ready cDNA (Clontech, Mountain View, CA) following manufacturer's instruction. Gene specific forward primers used were: mOfcc1-F1 (5′-CAGCAGAAGGCTGTGAAGCAAACTAAGCAG-3′ in exon 3), mOfcc1-F2 (5′-GTGAAGCAAACTAAGCAGAAGAAATCCACG-3′ in exon 3), and mOfcc1-F3 (5′-AGAAGAAATCCACGTCGGCTGAATTTCTGA-3′ in exon 3). Amplified fragments were cloned into the pCR4-TOPO vector (Invitrogen, Carlsbad, CA), and sequenced. Complimentary DNA corresponding to the full-length open reading frames for *Mrds1/Ofcc1* variants 1 and 2 were amplified by polymerase chain reaction (PCR) from mouse 15-day Marathon-ready cDNA (Clontech), and then cloned into the *Xho*I/*Sal*I site of the pAcGFP1-C1 vector (Clontech), to examine cellular localization of the protein.

### Generation of knockout mouse

All animal experiment protocols were approved by the Animal Experiment Committee of RIKEN Brain Science Institute and BioResouce Center. Mice were housed in groups of four or five in standard cages in a temperature and humidity controlled room, with a 12-h light/dark cycle (lights on at 08:00), and had access to standard lab chow and water *ad libitum*. The animals were used for behavioral tests from 3 to 4 months of age.

Major experimental tasks (design and construction of targeting vector, establishment of targeted ES cells, generation of the chimera and F1 mice, and removal of the neo cassette) to generate the *Mrds1/Ofcc1* knockout mice were performed by Unitech, Co. (Kashiwa, Japan). The knockout mice were produced on a background of C57BL/6. The founder mouse (+/Flox [neo+]) was crossed with the FLPe transgenic mouse, producing conditional knockout mice without the neo cassette (+/Flox [neo-]). Deletion of this cassette was confirmed by PCR. In this study, we created mice with a constitutionally deleted (KO) allele by crossing the +/Flox [neo-] mice with the CAG-Cre-Tg mouse, that systemically expresses Cre recombinase, under the control of a CAG promoter [21]. After backcrosses for at least four generations to C57BL/6N Slc (Japan SLC, Shizuoka, Japan), heterozygotes were intercrossed to obtain wild-type (WT), heterozygote and KO mice. Genotyping was performed by PCR with primer sets: (forward 1: 5′-CATGTGTACAGAACATGTGTGTAGC-3′, forward 2: 5′-AGCTCCTCTATTCTCTGGTTGAGG-3′, and reverse: 5′-AGGTAGAGGCTCCTC TGAGCTTAG-3′). Template DNA was extracted from tails or other tissues in some cases, producing 190 bp (forward 1 and reverse primers) and 270 bp (forward 2 and reverse primers) products from the WT and KO alleles, respectively.

To confirm the loss of *Mrds1/Ofcc1* mRNA transcripts, total RNA was extracted from E14.5 embryos using miRNeasy Mini (Qiagen, Hilden, Germany), and the first strand cDNA synthesized by oligo dT primer, was used as a template for RT-PCR. When necessary, nested (2nd) PCR was performed to detect specific bands. Primer pairs were: for the 1st PCR between the exons 3 and 4, 5′-GAGAAGTTTCAGCAGAAGGCTGT-3′ and 5′-ATGAGAAGAGCAGCAAGTGTGAG-3′, for the 2nd PCR between the exons 3 and 4, 5′-GAAGGCTGTGAAGCAAACTAAGC-3′ and 5′-GAGCACAATCAGGACTGAAGTCA-3′, for the PCR between the exons 3 and 6, 5′-GAAGGCTGTGAAGCAAACTAAGC-3′ and 5′-TGATGAAGAACCTGCAAGCAG-5′, and for the PCR between the exons 18 and 21, 5′-AAAGATCTGAGCAGTGGAGAGG-3′ and 5′-TTTCCACTCGGGTTAATGATCT-3′.

### Behavioral Analyses

Behavioral analyses were performed as described elsewhere [22], [23], [24]. All values in the text and figures represent mean ± SEM (n = 10–15/group). We analyzed the data using Student's *t*-test, and showed *P* values for comparisons between knockout animals and their control littermates. *P*<0.05 was considered significant. ANOVA (analysis of variance) followed by *post hoc* Tukey multiple comparison test was also used if required.

### Bone staining

Bone staining of E18.5 fetuses was performed as described [25], using Alzarin Red S (Sigma, St. Luis, MO) and Alcian Blue 8GS (Sigma, St. Luis, MO) for bone and cartilage, respectively.

### The Japan Mouse Clinic

The knockout mice were screened for abnormalities using systematic mouse abnormality screening called the ‘Japan Mouse Clinic’, a research system established and performed at the RIKEN BioResource Center (http://www.brc.riken.jp/lab/jmc/mouse_clinic/m-strain_jp.html). In this study, we analyzed the heterozygotes obtained by the intercross between heterozygous animals (backcrossed animals) as well as KO animals and WT littermates.

### Human DNA sample

All subjects were recruited from a geographic area located in central Japan. The family-based study samples consisted of 124 families with 376 individuals, of whom 163 subjects were schizophrenics and the remainder, unaffected. The types and numbers of families included 80 independent and complete trios (schizophrenic offspring and their parents), 15 probands with one parent, 13 probands with affected siblings, and 30 probands with discordant siblings. Best-estimate lifetime diagnosis was made by direct interview with at least two experienced psychiatrists and based on DSM-IV criteria, using all available information from medical records, hospital staff and family informants. Written informed consent was obtained from all participants and the study protocol was approved by the Committee on Ethics at RIKEN.

### Genotyping of human subjects and statistical analyses

The genomic structure of human *MRDS1/OFCC1* is based on Human Feb. 2009 (GRCh37/hg19) of the UCSC database (http://genome.ucsc.edu/) (Fig. S1). The single nucleotide polymorphism (SNP) search was performed using Entrez SNP from the NCBI database (http://www.ncbi.nlm.nih.gov/SNP/). We selected 32 SNPs within the *MRDS1/OFCC1* gene, including SNPs that showed strong evidence of association with schizophrenia in a prior study [8]. Twenty-five SNPs that could be unequivocally genotyped and showed sufficient heterozygosity (minor allele frequency >0.1) in our preliminary examinations were used for further analyses (Table S1 and Figure S1). *MRDS1/OFCC1* SNPs span a genomic stretch of 483,168 bp and the average marker-to-marker interval was ∼20 kb. These SNPs were selected to cover the *MRDS1/OFCC1* region as evenly as possible.

DNA was extracted from whole blood according to a standard protocol. We used Assays-by-Design SNP genotyping products to score SNPs (Applied Biosystems, Carlsbad, CA, USA) (http://www.appliedbiosystems.com/), based on the TaqMan assay methods. Genotypes were determined using an ABI7900 sequence detection system instrument (Applied Biosystems) and the SDS v2.0 software package (Applied Biosystems).

The normalized LD coefficient *D*′ [26] and squared correlation coefficient *r^2^* [27], parameters indicating a degree of LD between markers, were calculated, and haplotype structures were constructed, using HAPLOVIEW version 4.2 (http://www.broad.mit.edu/mpg/haploview/) [28]. Transmission distortions in the family panel were evaluated using the pedigree disequilibrium test (PDT) program, v5.1 (http://www.chg.duke.edu/research/software.html) [29]–[30]. The PDT program computes two statistical measures, PDT-sum and PDT-ave. Briefly, PDT-sum gives more weight to larger families, whereas PDT-ave places equal weight on all families. The suitability of both statistical methods depends on family structure and genetic models [29].

## Results

### Isolation of mouse *Mrds1/Ofcc1* transcripts

**Figure 1:**
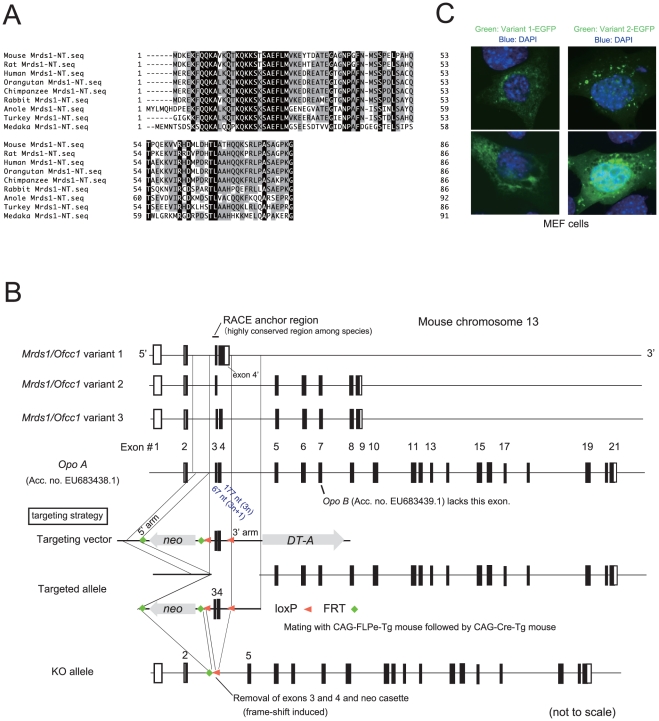
Identification of *Mrds1/Ofcc1* transcripts in mice and design of the targeting vector. **A**. Multiple alignment of Mrds1/Ofcc1 amino-acid sequences from different vertebrates. The homologous N-terminal regions of the Mrds1/Ofcc1/MRDS1/OFCC1 proteins were aligned using Genetyx-SV/RC (Ver. 15) software. **B**. Schematic view of the mouse *Mrds1/Ofcc1* gene structure. Structures of the three newly identified isoforms (variants 1, 2 and 3) are shown. White and black boxes show non-coding and coding exons, respectively. The structure of mouse *Opo A* is aligned with that of *Mrds1/Ofcc1.* The structure of the targeting vector, targeted and KO alleles are also shown. **C**. Cellular localization of Mrds1/Ofcc1-EGFP. MEF cells were transfected with plasmid expressing either variant 1-EGFP or variant 2-EGFP. EGFP signals (green) are shown with DAPI nuclear staining (blue). Two typical cells are shown for each variant: variant 1-EGFP protein localizes in a relatively diffuse manner within the cytoplasm (upper left panel) and, in part, as a net-like structure (lower left panel) in MEF cells. In contrast, the majority of variant 2-EGFP localizes as dot- or vesicle-like structures in the cytoplasm (upper right panel). Nuclear localization is also evident in the cells with strong expression of variant 2-EGFP (lower right panel).

The N-terminal region of the Ofcc1/Mrds1 protein is evolutionarily conserved among vertebrates including human, mouse, turkey and medaka fish (Figure 1*A*). However, the genomic structure of mouse *Mrds1/Ofcc1* is still unclear, particularly the 3′ region. We thus performed 3′-rapid amplification of cDNA ends (RACE) analysis with PCR primers placed in the conserved 5′ region. Three *Mrds1/Ofcc1* isoforms were isolated: variants 1, 2 and 3 (Figure 1*B*) with open reading frames (ORF) of 498 bp, 651 bp and 825 bp, respectively. The deduced amino-acid sequences of the 3 variants were not homologous to any known mouse proteins. Importantly, we found that while exons 2 through 8 of *Mrds1/Ofcc1* are shared with the mouse *Opo* gene [31], exons 1 (non-coding) and 9 were not (Figure 1*B*), suggesting that *Mrds1/Ofcc1* variants 1, 2 and 3 are shorter isoforms of the mouse *Opo* transcript.

Next, we examined cellular localization of Mrds1/Ofcc1 protein in mouse embryonic fibroblast (MEF) cells transfected with an expression vector for either variant 1 or 2 tagged with enhanced green fluorescent protein (EGFP) at the C-terminus. The variant 1-EGFP construct showed diffuse expression in the cytoplasm, in a partial net-like structure within MEF cells. In contrast, the majority of the variant 2-EGFP protein was localized as dot or vesicle like structures in the cytoplasm. Nuclear localization was also evident in cells with high expression of the variant 2-EGFP construct (Figure 1*C*).

### Generation of *Mrds1/Ofcc1* knockout (KO) mice

**Figure 2:**
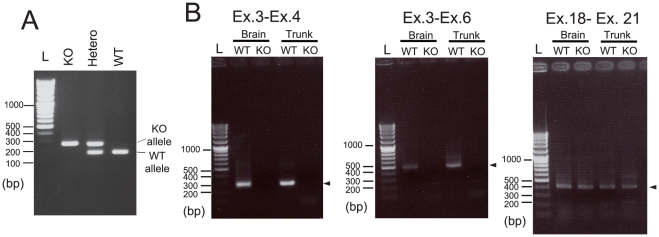
Transcript characterization of *Mrds1/Ofcc1* KO mice . **A**. Genotyping. Genotypes of the mice were determined by PCR. A typical gel image is shown. L: size marker **B**. Loss of mRNA for variants 1, 2 and 3, in the KO mice. Total RNA extracted from the E14.5 embryos (brain and trunk) was analyzed by RT-PCR and typical gel images are shown. Exon pairs in which specific primers were set are indicated above the gel images. Positions of specific bands are denoted by arrowheads. L: size marker.

We created two different types of KO mouse, systemic and conditional. In the conditional knockout alleles of the gene, exons 3 and 4 of the *Mrds1/Ofcc1* gene are flanked by two loxP sequences (Figure 1*B*). In this study, we analyzed the (systemic) KO mice, which were created by mating mice with conditional KO alleles with transgenic (Tg) mice expressing Cre recombinase (Figures 1*B* and 2*A*). The homozygous KO mice were born according to the Mendelian ratio and grew normally to adulthood (data not shown).

RT-PCR analysis using primer pairs designed between exons 3 and 4, and exons 3 and 6 showed a lack of transcripts for variants 1, 2 and 3 in the whole brain and trunk from embryonic day 14.5 (E14.5), KO embryos (Figure 2*B*, left and middle panels). As expected, mRNA expression in KO mice was detected with primer sets between exons 18 and 21 (Figure 2*B*, right panel). Deletion of exons 3 and 4, in all likelihood created out-of-frame (frame-shifted) transcripts (Figure 1*B*), producing biologically inactive proteins in the mutant mice.

### No craniofacial abnormalities in the KO mice

**Figure 3:**
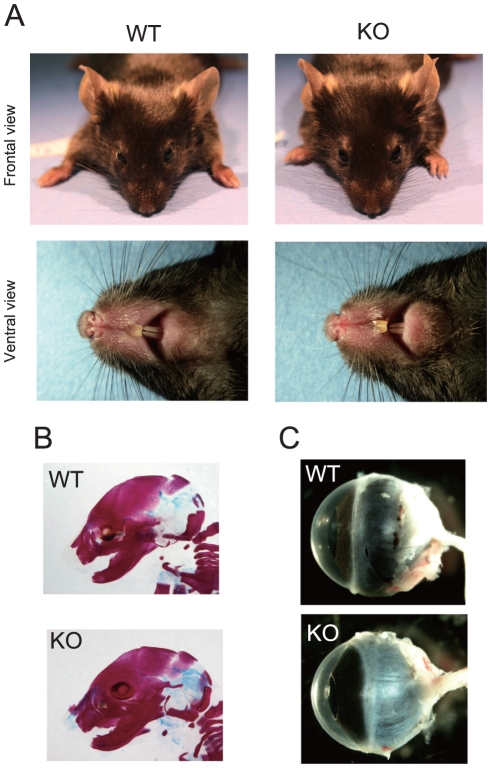
*Mrds1/Ofcc1*-deficient mice do not show any craniofacial or eye malformations. **A**. Facial appearance. Frontal (upper panels) and ventral views (lower panels) are shown. **B**. Head skull structure. E18.5 mice were sacrificed and stained with Alcian Blue (blue) and Alizarin Red (red) for cartilage and bone, respectively. **C**. Eyeball structure. Eyeballs were removed from adult mice and then photographed.

No orofacial clefts (cleft lip and/or cleft palate) were found in the greater than 50 examined mutant mice. There were no discernible differences between the typical appearance of WT and KO faces (Figure 3*A*). Nor did the head skull structures of the E18.5 KO fetuses differ from those of the control mice (Figure 3*B*).

The medaka *opo* mutant, where an ethylnitrosyrea (ENU) -induced mutation disrupts normal splicing of the *opo* gene, which is a potential ortholog for mouse and human *Mrds1/Ofcc1/MRDS1/OFCC1*, develops the *ojoplano* (‘flat eye’) phenotype due to defective eye cup morphogenesis [1]. No apparent eye abnormalities were observed in our KO mice (Figure 3 *A* and *C*), although we did not perform histological or immunohistochemical examinations. These data indicate that penetrance of the craniofacial abnormality generated by the deficiency of *Mrds1/Ofcc1* is, if existent, extremely low in mice.

### Genetic association of *MRDS1/OFCC1* with schizophrenia, but with no disease related behavioral abnormalities in KO mice

**Figure 4:**
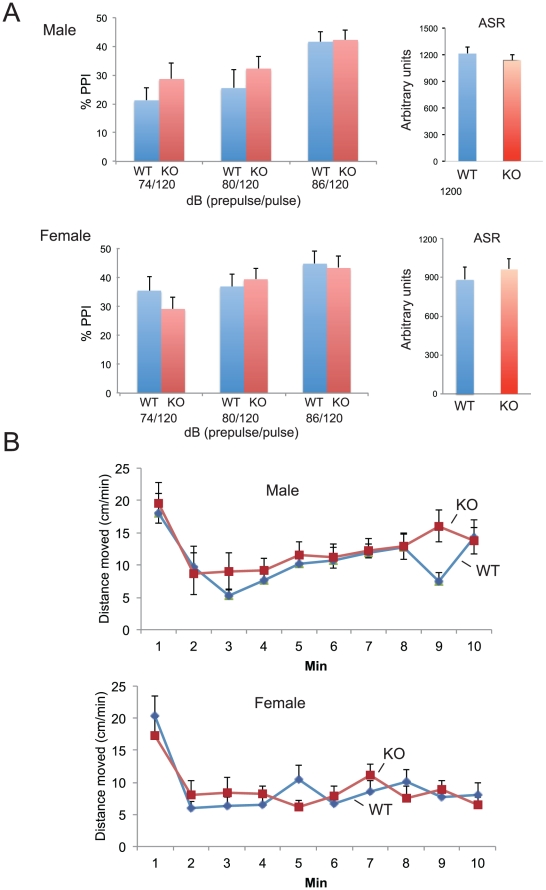
Behavioral tests for *Mrds1/Ofcc1* KO mice. **A**. Prepulse inhibition (PPI) test. Prepulse and pulse levels are shown under the X-axis. The acoustic startle response (ASR) at 120 dB is also shown (right). Blue and red bars show WT and KO mice, respectively. **B**. The open field test. Blue and red lines show WT and KO mice, respectively.

We examined whether the KO mice exhibited behavioral changes related to mental illnesses, including schizophrenia. We used a battery of tests consisting of open field, tail suspension, elevated plus maze, home cage activity, prepulse inhibition (PPI), and fear conditioning tests. Results are summarized in Table 1 and Figure *4*. Importantly, we detected no differences between WT and KO in PPI [two-way repeated ANOVA (prepulse level x genotype), genotype effect, male, *F*_(1,42)_  = 0.761, *P* = 0.388; female, *F*_(1,44)_  = 0.102, *P* = 0.751], a test of sensorimotor gating function (Figure 4*A*), although deficits in PPI have been reported in patients with schizophrenia. In addition, tests on our KO mice related to affection and cognition axes detected no behavioral changes which correlate with phenotypes seen in human mental disorders (Figure 4*B* and Table 1).

**Table 1:** Summary of behavioral analyses of *Mrds1/Ofcc1* KO mice.

		Male			Female		
Test battery		WT	KO	*P* (Student's *t*-test)	WT	KO	*P* (Student's *t*-test)
**OF**	Total distance (cm /10min)	3112.3±166.9	3490.5±212.4	0.1688	3547.2±203.4	3881.7±149.1	0.1916
	Center time (cm /10 min)	104.8±14.9	124.1±16	0.3824	90.7±7.0	90±11.6	0.959
**EPM**	Open arm stay time (%)	17.8±3.5	17.6±4.9	0.9737	19.4±4.7	29.6±5.0	0.1443
	Closed arm stay time (%)	82.3±3.5	82.5±4.9	0.9737	80.7±4.7	70.5±5.0	0.1443
	Total distance (cm)	738.7±42.9	770.0±44.4	0.6148	753.2±49.4	838.1±57.6	0.2693
**LD**	Stay time (L) (sec/10 min)	529.0±9.6	531.5±6.9	0.8335	548.2±8.8	534.1±10.2	0.301
	Stay time (D) (sec/10 min)	81.0±9.7	78.5±7.4	0.8377	61.3±9.0	75.1±10.4	0.3212
	Transition numbers	14.0±1.7	13.7±1.5	0.8954	10.7±1.6	12.0±1.6	0.5685
	Latency (D to L) (sec/10 min)	112.7±20.9	124.3±21.5	0.7008	109.6±21.1	100.0±24.3	0.7669
**PPI**	74/120 dB %	21.3±4.5	28.8±5.5	0.388*	35.5±4.9	29.2±4.2	0.751*
*(prepulse level/pulse level)*	80/120 dB %	25.6±6.4	32.4±4.2		37.0±4.3	39.5±3.8	
	86/120 dB %	41.7±3.5	42.4±3.5		44.9±4.4	43.4±4.2	
	ASR	1212.1±72.6	1137.8±60.3	0.4338	884.2±94.0	965.7±79.2	0.5108
**FST**	Immobility time (sec/5min)	146.0±9.1	130.6±8.2	0.211	149.1±8.3	164.2±7.7	0.1892
**TST**	Immobility (%)	15.4±3.1	18.2±2.4	0.5341	25.8±6.6	18.7±4.0	0.3626
**HCA**	Locomotor activity (x10^4^)	7.6±0.6	7.4±1.5	0.8947	9.2±1.9	12.3±1.1	0.1814
**FC**	Conditioning (freezing %)	29.2±3.0	22.6±2.4	0.0916	16.0±1.6	14.9±1.8	0.6501
	Contextual test (freezing %)	71.6±4.0	63.8±4.0	0.1752	52.6±4.1	58.7±4.0	0.2927
	Cued test (freezing %)	62.4±3.2	62.6±3.9	0.9686	48.4±4.4	54.9±4.2	0.2911

OF; the open field test; EPM: elevated puls maze test; LD: light and dark box transition test; PPI: prepulse inhibition test; FST: forced swim test; TST: tail suspension test; HCA: home cage activity test; FC: fear conditioning test; ASR: acoustic startle response without prepulse. *Statistical analysis for PPI was performed by two-way repeated ANOVA between multiple prepluse levels and genotypes. All values represent mean ± SEM (n = 10–15/group).

In contrast to results from animal studies, we found an association between schizophrenia and *MRDS1/OFCC1* in a Japanese cohort. Five SNPs that span a 197 kb region of the gene (rs7741209–rs855394), showed significant transmission distortion in a family-based association study (lowest *P* = 0.0082 for rs7741209) (Figure S1 and Table S1). Pairwise marker-to-marker linkage disequilibrium (LD) and haplotype block structures based on the standard Lewontin *D*′ statistic and squared correlation coefficient *r^2^* statistic are shown (Figure S2, *A* and *B*). The SNPs that showed significant association with schizophrenia were not in tight LD to each other (Figure S2, *A* and *B*). This may reflect an allelic heterogeneity of the gene that confers susceptibility to schizophrenia in the Japanese population.

### Significant increase in serum γ-glutamyl transpeptidase (GGT) levels in KO mice

**Figure 5:**
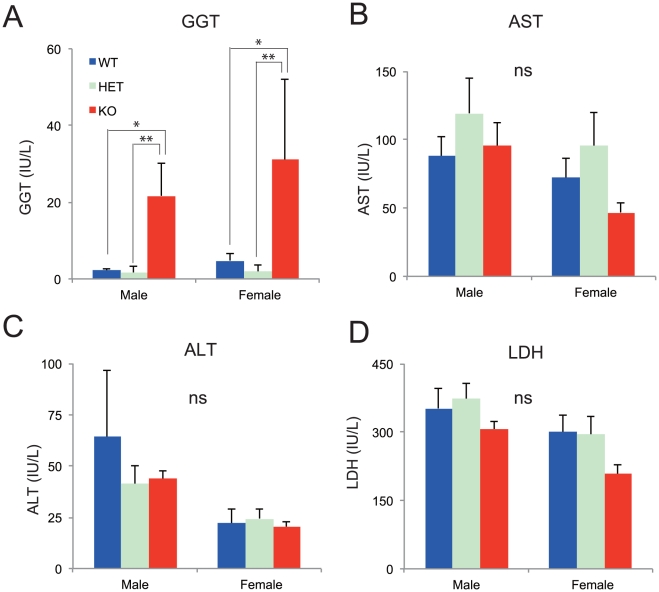
Significant increase in γ-glutamyl transpeptidase expression in KO mice. **A**, **B**, **C**, and **D**. Liver-related measures derived from serum examined in the Japan Mouse Clinic are shown in **A** (GGT: γ-glutamyl transpeptidase), **B** (AST: asparagine transferase), **C** (ALT: alkaline phosphatase), and **D** (LDH: lactic dehydrogenase). ns, not significant. ^*^ *P*<0.01, ^**^ *P*<0.001 by Tukey multiple comparison.

To obtain insight into the biological function of *Mrds1/Ofcc1*, we analyzed the KO mice using the ‘Japan Mouse Clinic’, a comprehensive and systematic abnormality screening system conducted by the RIKEN BioResource Center [32] (http://www.brc.riken.jp/lab/jmc/mouse_clinic/m-strain_jp.html). This system analyzes mice in a two-step manner, via pipeline 1 and pipeline 2. Tests in pipeline 1 are categorized into two groups, fundamental and in-depth screens. The fundamental screen is performed using six methods: the open field test, modified SHIRPA (SmithKline Beecham Pharmaceuticals; Harwell, MRC Mouse Genome Centre and Mammalian Genetics Unit; Imperial College School of Medicine at St Mary's; Royal London Hospital, St Bartholomew's and the Royal London School of Medicine; Phenotype Assessment), hematological tests, urinalysis, clinical biochemical tests and a macroscopic test. The in-depth screen is performed using 11 methods: rotarod test, auditory brain stem response (ABR), insulin tolerance test (ITT), oral glucose tolerance test (OGTT), lactate measurements in blood, adipokines and clinical biochemical tests (glucose and adipose related), fundoscopy, ERG (electroretinography), blood pressure, body fat percentage, bone mineral density (by DEXA; dual-energy X-ray absorptiometry) and echocardiography. The extensive pipeline 1 screen revealed that two measures were significantly different among the genotypes: γ-glutamyl transpeptidase (GGT) levels (Figure 5*A*) and ‘tail ratio’ (a ratio of body length to tail length) [Figure S3, WT vs. heterozygote (HET), *P* = 0.02 by Tukey multiple comparison]. Levels of GGT, which are measured in the ‘clinical biochemical test (lipid and glucose)’, were markedly higher in KO mice compared to WT and HET mice of either sex [Two-way ANOVA (genotype x sex), genotype effect, *F*_(2,44)_  = 10.449, *P* = 0.00019; *post hoc* Tukey multiple comparison, HET vs. KO, *P* = 0.00034, WT vs. KO, *P* = 0.0011] (Figure 5*A*).

GGT is a reliable and established marker for liver function in human clinical practice. However, we did not find any other markers suggesting liver dysfunction in serum or (Figure 5, *B*-*D*) in liver tissue (data not shown). In humans, elevated levels of GGT are seen in the early phase of alcoholic hepatopathy. Additionally, human kidney and pancreas are GGT enriched tissues, but we found no evidence suggesting abnormalities of these two organs in the KO mice (data not shown). Therefore, it remains unclear why the KO mice have notably increased serum levels of GGT. To our knowledge, this is the first report of asymptomatic hyper-γ-glutamyl transpeptidasemia in mice.

Pipeline 2 is a behavior-oriented screen, consisting of light/dark transition, open-field, home-cage activity, passive avoidance, tail suspension, hot plate and tail flick tests. We detected no significant differences among the three genotypes, WT, HET and KO in these tests. The optional three tests, PPI, object exploration and fear conditioning tests were not performed.

## Discussion

The *MRDS1/OFCC1/Mrds1/Ofcc1/Opo* gene is structurally conserved among vertebrates, suggesting a phylogenically important role for this gene. In this study, we created and analyzed *Mrds1/Ofcc1* KO mice, where the gene construct lacks exons 4 and 5, generating a frame-shift mutation in the mRNA and thereby producing a premature termination codon that potentially abolishes the biological activity of the protein. Mice were analyzed thoroughly using amongst other tests, the Japan Mouse Clinic, a systematic abnormality screening system for mutant mice. Despite its genetic involvement in craniofacial and eye development and the pathogenesis of schizophrenia, no discernible abnormalities in mutant mice were detected, except for surprisingly high levels of GGT in the sera. A possible explanation for these results is that an as yet unidentified gene with structural homology to *Mrds1/Ofcc1* compensates for the loss of the *Mrds1/Ofcc1* at a functional level. This is however unlikely, as an extensive homology search of the database by researchers including ourselves, has failed to identify any gene homologous to *Mrds1/Ofcc1* in the mouse genome.

In medaka fish, a hypomorphic mutation in *Opo,* results in multiple malformations of craniofacial structures, including the eyes. It is possible that the function of mammalian *Mrds1/Ofcc1/MRDS1/OFCC1* differs from that of medaka *Opo*. It is also possible that the genetic background of mice may influence the penetrance of the predicted eye phenotypes. Alternatively, one or more biologically active, uncharacterized transcripts that do not include exons 4 and 5 may be present. In this study, we detected a transcript containing exons 18 through 21, in the KO mice (Figure 2*B*, right panel). This scenario seems unlikely as medaka opo proteins are expressed as two isoforms of 42 and 130 kDa [1], and a hypomorphic mutation of the gene results in a reduction of the two proteins.

As stated earlier, *OFCC1* is also a positional candidate gene for orofacial cleft (*OFC1*, OMIM119530). However, we detected no abnormalities in craniofacial development in the KO mice. Although we cannot rule out the possibility that abrogated *MRDS1/OFCC1*/*Mrds1/Ofcc1* is insufficient to cause craniofacial abnormalities, the simplest explanation for the current observation is that *MRDS1/OFCC1* is not a causal gene for orofacial cleft seen in subjects with a chromosomal break at 6p24. Consistent with this idea, is the identification of the other candidate gene for *OFC1* locus, *TFAP2A* [2] which lies proximal to *MRDS1/OFCC1*. While this gene does not appear to be directly disrupted by chromosomal breaks in the three affected subjects, *Tfap2a* knockout mice exhibit pleiotropic morphological abnormalities, including orofacial cleft [33].

Interestingly, our KO mice exhibited strikingly high levels of GGT in sera. This phenotype of high but asymptomatic levels of GGT has not been reported previously in mammals. None of the examined schizophrenic offspring showed extremely high levels of GGT in the absence of other markers for tissue damage. Discovery of this asymptomatic, high γ-glutamyl transpeptidasemia phenotype would have been difficult, without the availability of the Japan Mouse Clinic, a research tool established by our institute. This discovery could assist in identifying corresponding human subjects in a clinical setting. Examining the relationship between schizophrenia and liver function-related parameters, some patients with schizophrenia manifest asymptomatic hyperbilirubinemia [34]. However, our KO mice showed normal levels of bilirubin. Recent studies have found the relationship GGT levels and cardiovascular diseases independent on the liver function, although we found no sign for cardiovascular defect in the KO mice [35], [36].

In this study of KO mice, we were unable to identify any schizophrenia-relevant behavioral abnormalities. It may be interesting to examine how the behavior of the KO mice is modulated when they are placed under stress-loaded conditions such as separation of pups from dams or administrated with psychostimulants like methamphetamine. In the human study, we have found evidence of a genetic association between the *MRDS1/OFCC1* gene and schizophrenia in a Japanese sample. To corroborate this association, further studies using larger sample sizes will be needed.

Finally, future analysis of our *Mrds1*/*Ofcc1*-KO mice could be valuable in deciphering the mechanism of high γ-glutamyl transpeptidasemia and its possible relationship to schizophrenia.

## Supporting Information

Figure S1 **Genomic structure of the** ***MRDS1/OFCC1*** **gene and gene-centric association analysis.** Genomic structure of the *MRDS1/OFCC1* gene is shown with chromosomal positions according to the human genome database (http://genome.ucsc.edu/). The negative logarithm of *P* value for association is plotted as a function of the order of SNP markers. Statistical analyses were performed using the pedigree disequilibrium test. Red diamond-shaped marks denote SNPs with *P*<0.05, while blue marks denote SNPs with *P*>0.05.
(EPS)

Figure S2 **Pairwise marker-to- marker linkage disequilibrium structure in Japanese samples.** (**A** and **B**) The standard Lewontin *D*′ statistic (**A**), and the squared correlation coefficient *r^2^* statistic (**B**) are shown. Bold SNPs are those associated with schizophrenia.
(EPS)

Figure S3 **The ‘tail ratio’ in knockout mice.**
(EPS)

Table S1 **Summary of genetic association study.** SNP ID: Entrez SNP database (http://www.ncbi.nlm.nih.gov/snp) Position: Based on the UCSC Genome Browser on Human Feb. 2009 (GRCh37/hg19) Assembly (http://genome.ucsc.edu/) SUM: PDT-SUM statistics, AVE: PDT-AVE statistics.
(DOC)
