# Supplementary material for: Ablation of Mrds1/Ofcc1 Induces Hyper-γ-Glutamyl Transpeptidasemia without Abnormal Head Development and Schizophrenia-Relevant Behaviors in Mice
Source: PLoS One. 2011 Dec 29;6(12):e29499. doi: 10.1371/journal.pone.0029499 (PMC3248446; doi:10.1371/journal.pone.0029499)
Supplement: Table S1 — Summary of genetic association study. SNP ID: Entrez SNP database (http://www.ncbi.nlm.nih.gov/snp) Position: Based on the UCSC Genome Browser on Human Feb. 2009 (GRCh37/hg19) Assembly (http://genome.ucsc.edu/) SUM: PDT-SUM statistics, AVE: PDT-AVE statistics. (DOC) [file pone.0029499.s004.doc]

**Supplemental Table S1**

Summary of genetic association study

| SNP ID | Position | PDT (*P* value) | |
| --- | --- | --- | --- |
|  |  | SUM | AVE |
| rs3918445 | 9582818 | 0.3701 | 0.4047 |
| rs9396479 | 9655991 | 0.3232 | 0.3876 |
| rs1206914 | 9770501 | 0.2159 | 0.4882 |
| rs1206963 | 9831493 | 0.0516 | 0.1629 |
| rs7741209 | 9852464 | ***0.0082*** | ***0.0147*** |
| rs2096022 | 9878990 | 0.5716 | 0.3219 |
| rs10949373 | 9892068 | 0.1892 | 0.2364 |
| rs1925767 | 9906496 | 1.0000 | 0.7024 |
| rs9358107 | 9907374 | 0.1032 | 0.1863 |
| rs2068317 | 9916994 | ***0.0246*** | 0.1419 |
| rs1925772 | 9921574 | ***0.0187*** | 0.1110 |
| rs2226048 | 9929099 | 0.6394 | 0.9120 |
| rs1022921 | 9944678 | ***0.0187*** | 0.1110 |
| rs201267 | 9974873 | 1.0000 | 0.5781 |
| rs201256 | 10004376 | 1.0000 | 0.8685 |
| rs201253 | 10007456 | 0.4795 | 0.9152 |
| rs201227 | 10028792 | 0.8997 | 0.8299 |
| rs1322827 | 10033227 | 0.4452 | 0.9542 |
| rs707783 | 10040254 | 0.3248 | 0.6162 |
| rs763908 | 10041490 | 0.0561 | 0.2763 |
| rs2179365 | 10045974 | 0.3272 | 0.9864 |
| rs855394 | 10049717 | ***0.0477*** | ***0.0447*** |
| rs1322823 | 10056122 | 0.7316 | 0.9257 |
| rs760892 | 10058811 | 0.1436 | 0.3152 |
| rs1407621 | 10065985 | 0.1742 | 0.3797 |

SNP ID: Entrez SNP database (http://www.ncbi.nlm.nih.gov/snp)

Position: Based on the UCSC Genome Browser on Human Feb. 2009 (GRCh37/hg19) Assembly (http://genome.ucsc.edu/)

SUM: PDT-SUM statistics, AVE: PDT-AVE statistics
